# Value of self-performed joint counts in rheumatoid arthritis patients near remission

**DOI:** 10.1186/ar3777

**Published:** 2012-03-14

**Authors:** Helga Radner, Johannes Grisar, Josef S Smolen, Tanja Stamm, Daniel Aletaha

**Affiliations:** 1 Department of Internal Medicine III, Division of Rheumatology, Medical University of Vienna, Waehringerguertel 18-20, Vienna, 1090, Austria

## Abstract

**Introduction:**

To determine the validity and reliability of patients' self-performed joint counts compared to joint counts by professional assessors in rheumatoid arthritis (RA) patients in different disease activity states.

**Methods:**

In patients with established RA we determined the inter-rater reliability of joint counts performed by an independent evaluator and the patient using intraclass correlation (ICC), and agreement on activity in individual joints by kappa statistics. We also performed longitudinal analyses to assess consistency of assessments over time. Finally, we investigated the concordance of joint counts of different assessors in patients with different levels of disease activity.

**Results:**

The reliability of patient self-performed joint counts was high when compared to independent objective assessment (ICC; 95%confidence interval (CI)) for the assessment of swelling (0.32; 0.15 to 0.46) and tenderness (0.75; 0.66 to 0.81), with higher agreement for larger joints (kappa: 0.57 and 0.45, respectively) compared to smaller joints (metacarpo-phalangeal joint (MCPs): 0.31 and 0.45; and proximal interphalangeal joint (PIPs): 0.22 and 0.47, for swelling and tenderness, respectively).

Patients in remission according to the Simplified Disease Activity Index (SDAI ≤ 3.3) showed better concordance of the joint counts (swollen joint count (SJC) ties 25/37, tender joint count (TJC) ties 26/37) compared to moderate/high disease activity states (SDAI > 11; MDA/HDA: SJC ties 9/72, TJC ties 21/72). Positive and negative predictive values regarding the presence of SDAI remission were reasonably good (0.86 and 0.95, respectively). A separate training session for patients did not improve the reliability of joint assessment. The results were consistent in the longitudinal analyses.

**Conclusions:**

Self-performed joint counts are particularly useful for monitoring in patients having attained remission, as these patients seem able to detect state of remission.

## Introduction

Rheumatoid arthritis is a chronic disease characterized by an inflammatory process which, over time, leads to irreversible joint destruction. This joint damage is unequivocally related to the clinical involvement of the joints [1,2]; therefore, assessment of joint involvement by examining their swelling and tenderness is crucial in RA. Formal joint assessments, so called joint counts, are generally performed by physicians, nurses or other health professionals. Measures of joint activity regarding swelling and tenderness are also part of most composite disease activity indices in RA.

In recent years, formal disease activity assessment has moved into the focus of RA management [3] and new treatment strategies have been brought forward suggesting that remission may be one of the main targets in treating RA [4]. The state of remission is superior to other disease activity states, including low disease activity, with regard to structural, functional, and economic outcomes [5,6]. This is also reflected in the 2010 European League against Rheumatism (EULAR) management recommendations for RA, which suggest constant evaluations and rapid treatment adaptations until the goal of remission is reached [4].

However, a close and accurate assessment of disease activity is time consuming and, as many clinics are facing limited outpatient capacity, physicians are often reluctant to perform complete joint counts and are also forced to reduce the frequency of their patients' routine visits once their arthritis has improved. Also, since the ultimate goal is not only reaching but rather maintaining remission for long term, it is necessary to monitor patients closely to control for persistence of remission. Nevertheless, candidates for reductions in outpatient contacts would be patients in remission rather than those with active disease who, from a medical perspective, require more intensive attention. This is also reflected in the treat-to-target recommendations [7] where it is suggested that patients with active disease should be seen monthly while for patients in remission control examinations are recommended every six months or less frequently. However, these patients still require monitoring of disease activity, since the risk of flares remains, even while continuing therapy [8]. Although the term flare has not yet been well defined, the recurrence of clinical joint activity is most likely an indicator of such a flare. Patients may be scheduled for a clinic or office visit at another time point than that around recurrence of inflammatory joint activity, and the time elapsing until that scheduled visit may allow for significant damage to occur or may make the flare more difficult to manage. However, if patients could monitor their own joints for swelling, they could alert their rheumatologist for an immediate clinic appointment.

Several studies have addressed patient self assessed swollen and tender joint counts as well as their correlation with evaluator derived joint counts [9]; the results of these studies are controversial, partially concluding that patient derived joint counts are simply not accurate enough. None of these studies, however, looked separately at patients in different states of disease activity, especially in remission.

In the present study, we, therefore, were interested to evaluate the reliability of self-assessed joint counts among RA patients, but aimed to focus on patients in different disease activity states including remission to investigate if sensitivity for joint activity of self-performed joint counts changes with the extent of disease activity. We hypothesized that the reliability of self-assessed joint counts would increase with decreasing disease activity which, if proven, would allow scheduling RA patients' visits in remission at longer intervals, while they could monitor the maintenance of remission on a more frequent basis at home. We also investigated whether simple training sessions might improve this sensitivity.

## Materials and methods

### Study design

A total of 209 consecutive RA patients, routinely visiting our outpatient department were randomly recruited into the study; all patients who were included provided informed consent. No further inclusion or exclusion criteria applied. The study was approved by the Ethics Committee of the Medical University of Vienna.

Traditional 28-joint counts were performed for swelling and tenderness by the patient, a biometrician, and a rheumatologist. After the participants had performed and documented their self- assessment, tender and swollen joint counts were determined by a biometrician, a trained health professional performing joint counts regularly at our clinic, and a rheumatologist, who was not involved in treatment decisions. Patients, biometrician, and the physician were blinded to each other's results, and patients were advised not to discuss their assessment with the other assessors. In addition, routine clinical and laboratory measurements, such as the health assessment questionnaire (HAQ), C-reactive protein (CRP) and erythrocyte sedimentation rate (ESR), were also collected; composite indices, such as the clinical disease activity index (CDAI), simplified disease activity index (SDAI) and disease activity score using 28 joints (DAS28), were calculated. The biometrician's joint counts were used as the gold standard, since their assessment is the documented routine procedure in our clinic. The whole process was repeated at a subsequent clinical visit three months later, to allow assessment of longitudinal reliability.

### Patient instructions

**Figure 1:**
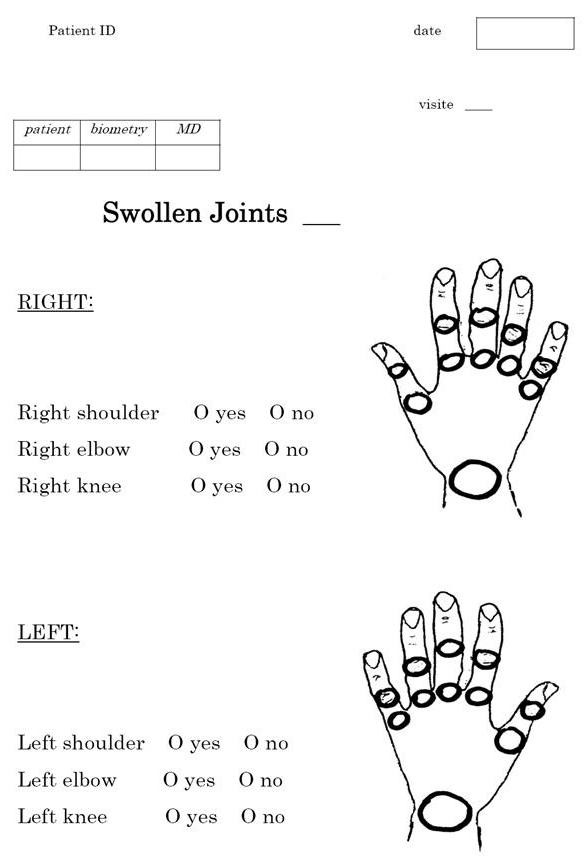
**Joint count questionnaire for swelling, similar questionnaire was filled in for tenderness**.

Patients were pseudo-randomly allocated to two groups in a 2:1 manner: consecutive patients who gave informed consent visiting our department on odd calendar days received formal joint count training by a physician, whereas patients visiting on even days remained untrained. Those on even days (*n *= 131) were merely instructed to mark the tender and swollen joints in two respective drawings presenting 28 joints, based on their own joint assessment for swelling and tenderness (Figure 1). On odd days (*n *= 78) patients received a short training session with a special emphasis on the explanation of joint swelling in terms of soft tissue (synovial) versus bony swelling. Afterwards patients were instructed to put their marks on the simple, structured questionnaires. The randomization process was performed by the first author who was not involved in the assignment of visit date.

### Cross sectional analysis

Two-way comparisons of the joint count results obtained by the three assessors were performed using an intraclass correlation coefficient (ICC) for the count (0 to 28), as well as Kappa statistics for each individual joint and joint region. To assess the value of the structured training session, we also compared correlations of trained and untrained patients using ICC to determine whether simple training can improve the correlation between patient and independent assessor.

### Longitudinal analysis

Longitudinal assessments were used to determine whether changes in joint counts observed over the three month period were consistent among the different assessors (correlation of changes) and to test whether patients improve their ability to assess joint swelling and tenderness (Wilcoxon-Test).

### Validity of self assessed joint counts in different disease activity states

Next, patients were divided according to the levels of disease activity by SDAI and inter-rater-reliability was assessed in three different subgroups, remission (REM, SDAI < 3.3), low disease activity (LDA, 3.3 < SDAI < 11) and moderate to high disease activity (MDA/HDA, SDAI > 11). We then repeated the cross sectional analyses as outlined above in the three subgroups to assess differences according to disease activity levels.

### Validity of self assessed joint counts in determining the presence of remission

Concordance of remission rates was compared using either patient self-assessed joint counts or joint counts performed by a physician or biometrician. Remission was defined according to new American College of Rheumatology (ACR)/EULAR Remission Criteria [7] using the Boolean criteria as swollen joint count (SJC) < 1, tender joint count (TJC) < 1, patient global assessment of disease activity (PGA) < 1 cm with and without CRP < 1 mg/dl and the index-based criteria, SDAI (≤ 3.3) and CDAI (≤ 2.8).

This study was approved by the Ethics Committee of the Medical University of Vienna. The Statistical Package for the Social Sciences (SPSS, Version 17) was used for the conduct of the analyses.

## Results

In total, our cohort comprised 209 patients with established RA, who were randomly selected at our clinic. Baseline characteristics are shown in Table 1. Regarding treatment strategies, in total 4.8% did not receive any disease modifying anti-rheumatic drug (DMARD) due to different reasons (remission, pregnancy), 63.6% of patients received synthetic DMARDs, and 31.6% received biological agents with or without synthetic DMARDs. We found no significant differences of distribution of treatment strategies within trained and untrained patients or within different levels of disease activity.

**Table 1:** Baseline characteristics of included patients.

				*P*-value
Baseline characteristics	TOTAL	TRAINED	UNTRAINED	trained vs. untrained
Number	209	78	131	
Female	80.4%	79.5%	80.9%	
Rheumatoid factor pos.	55.5%	52.6%	57.3%	
Age (years)	56.3(12.3)	54.2 (12.6)	57.6 (12.0)	0.06
Disease duration (years)	11.4 (9.6)	11.0 (9.2)	11.6 (9.9)	0.66
PGA (mm)	28.1 (21.9)	28.1 (22.4)	28.0 (21.7)	0.97
VAS pain (mm)	26.2 (21.0)	26.1 (20.9)	26.2 (21.2)	0.99
EGA (mm)	12.9 (13.5)	16.3 (15.7)	10.8 (11.5)	0.004
CRP (mg/dl)	0.92 (2.0)	1.0 (1.7)	0.89 (2.2)	0.81
ESR (mm/hr)	26.2 (22.3)	28.2 (26.1)	25.1 (20.1)	0.38
HAQ	0.78 (0.72)	0.81 (0.74)	0.77 (0.71)	0.71
SJC28 biometrican	2.4 (3.0)	3.3 (3.3)	1.9 (2.7)	0.001
TJC28 biometrican	2.7 (4.5)	2.4 (4.1)	2.9 (4.7)	0.48
SJC28 physician	2.4 (1.9)	3.1 (3.1)	1.9 (2.7)	0.004
TJC28 physician	3.0 (4.1)	2.9 (3.7)	3.0 (4.4)	0.85
SJC28 patient	3.1 (4.3)	2.7 (3.6)	3.4 (4.7)	0.28
TJC28 patient	3.4 (5.2)	3.0 (4.5)	3.7 (5.6)	0.33
CDAI	9.0 (7.4)	10.1 (7.9)	8.4 (7.0)	0.097
SDAI	10.0 (7.8)	11.1 (8.5)	9.3 (7.4)	0.094
DAS28	3.4 (1.1)	3.4 (1.2)	3.4 (1.1)	0.33

Data are shown as mean (SD) unless indicated otherwise.

CDAI: Clinical Disease Activity Index, CRP: c-reactive protein, DAS28: disease activity score 28, EGA: evaluator global assessment of disease activity, ESR: erythrocyte sedimentation rate, HAQ: health assessment questionnaire, PGA: patient global assessment of disease activity, SDAI: simplified disease activity score, SJC28: swollen joint count, TJC28: tender joint count, VAS: pain visual analogue scale of pain

### Cross sectional analysis of inter-rater variability

Using ICC we found significant consistency of patient derived joint counts with those of the two independent observers (*P *< 0.003) (Table 2) at both visits.

**Table 2:** Intraclass correlation of different observer derived joint counts at baseline and follow up visit.

	Biometrician versus Patient		Physician versus Patient	
	
	Biometrician ICC (95% CI)		Independent physician ICC (95% CI)	
**Patient derived Joint counts**	**Baseline**	**Follow-up**	**Baseline**	**Follow-up**

***TOTAL***	***N = 209***	***N = 144***	***N = 209***	***N = 144***

Swollen Joint Count 28	0.30 (0.18- 0.42)	0.38 (0.23-0.51)	0.35 (0.22-0.46)	0.43 (0.28-0.55)
Tender Joint Count 28	0.70 (0.62-0.76)	0.71 (0.62-0.79)	0.80 (0.74-0.84)	0.82 (0.76-0.87)

***UNTRAINED PATIENTS***	***N = 131***	***N = 87***	***N = 131***	***N = 87***

Swollen Joint Count 28	0.32 (0.15-0.46)	0.33 (0.13-0.51)	0.39 (0.23-0.52)	0.41 (0.22-0.57)
Tender Joint Count 28	0.75 (0.66-0.81)	0.72 (0.60-0.81)	0.85 (0.80-0.90)	0.81 (0.72-0.87)

***TRAINED PATIENTS***	***N = 78***	***N = 57***	***N = 78***	***N = 57***

Swollen Joint Count 28	0.35 (0.14-0.53)	0.50 (0.28-0.67)	0.33 (0.12-0.51)	0.48 (0.25-0.66)
Tender Joint Count 28	0.59 (0.42-0.72)	0.72 (0.56-0.82)	0.66 (0.51-0.77)	0.84 (0.74-0.90)

**Physician derived Joint counts**	**Baseline**	**Follow-up**		

Swollen Joint Count 28	0.95 (0.93-0.96)	0.90 (0.87-0.93)	1	1
Tender Joint Count28	0.86 (0.82-0.89)	0.89 (0.85-0.92)	1	1

Consistency of physician and biometrician derived joint counts served as a benchmark for the interpretation of the validity of patient derived joint counts, showing high concordance (*P *< 0.001). Due to the high concordance of physician and biometrician joint counts, we used the biometrician derived joint counts as the gold standard reference.

### Effect of the structured patient training session

Comparing the correlations of patient and observer derived joint counts between the trained and the untrained patient groups, we found no noteworthy differences: looking at the baseline assessments, in the untrained group the ICC was 0.32 (0.15 to 0.46) for SJC28 and 0.75 (0.66 to 0.81) for TJC28; for the trained group, the ICC was 0.35 (0.14 to 0.53) for SJC28 and 0.59 (0.42 to 0.72) for TJC28. Similar results were obtained for the follow up visit (data not shown).

As a consequence of these data, we pooled the two groups of trained and untrained patients for the more detailed analyses to follow.

### Validity of patient self-assessed joint counts by joints and joint regions

**Figure 2:**
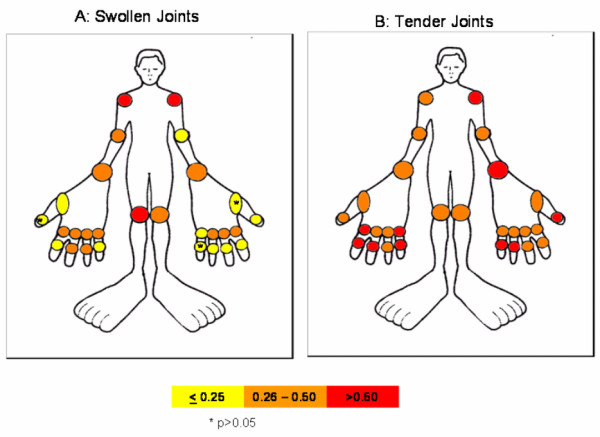
**Correlation between patient and biometrician derived joint counts showing kappa values of individual joints**.

Using Kappa statistics in all 209 patients, we analyzed the agreement between the two assessors (patient and biometrician) at the baseline visit by individual joints and joint region. A non-significant agreement was found for three small joints (left metacarpophalangeal joint I (MCP I), κ = 0.13; left proximal interphalangeal joint V (PIP V), κ = 0.13; right interphalangeal joint (IP), κ = 0.05). In general the small joints of the right hand tended to show better correlation with the biometrician derived joint count compared with those of the left hand (mean kappa right hand = 0.31; left hand = 0.23; T-test *P *= 0.09) (Figure 2). Kappa also differed when calculated for the various joint regions or types: 0.23 for swelling of large joints (shoulders, knees and elbows), 0.45 for tenderness of large joints; 0.31 and 0.45 for swelling and tenderness of MCPs, respectively; 0.22 and 0.47 for PIPs; and 0.31 and 0.45, respectively, for swelling and tenderness of the total hand (MCPs, PIPs and wrists). In summary, we found a consistently superior agreement of joint tenderness compared to joint swelling.

### Follow up assessment and longitudinal analyses

Three months after the initial visit, 144 of the 209 patients (69%; 57 trained, 87 untrained) were re-assessed for their joint activity in a similar manner. We found significant consistency between patient and observer joint counts (again biometrician data were used; Table 2) at the follow up visit compared to the baseline visit: SJC 0.38 (0.23 to 0.51) versus 0.30 (0.18 to 0.42); TJC 0.71 (0.62 to 0.79) versus 0.70 (0.62 to 0.76).

To investigate whether discrepancies in joint assessment were consistent over time we used ICC, correlating differences between patient and observer derived joint counts at baseline with those at follow up; these analyses showed that the discrepancy was similar over time, that is, a good correlation of discrepancy at the two time points, ICC (95% confidence interval (CI)) SJC = 0.56 (0.44 to 0.66); TJC = 0.36 (0.21 to 0.50) (*P *< 0.001).

We further used ICC calculations to compare whether changes of joint counts assessed by patients were concordant with changes observed by the independent assessor. This showed a low but significant (*P *= 0.02) correlation of change of SJC (ICC = 0.17; 0.01 to 0.33) and TJC (ICC = 0.35; 0.2 to 0.48; *P *< 0.01).

### Validity of self assessed joint counts in different disease activity states

**Figure 3:**
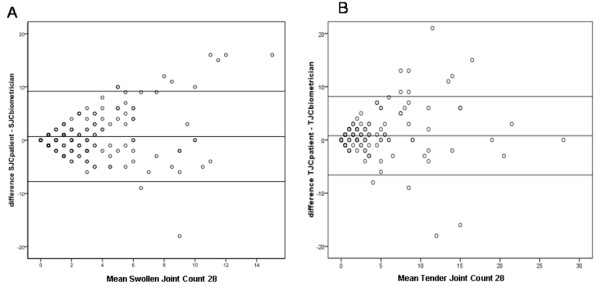
**Discordance of joint assessment is dependent on overall number of active joints**. Bland-Altman-Plot for patient and biometrician joint counts, plotting the differences of their joint counts (y-axis) against the mean of the joint counts (x-axis). The discordance of assessment increases with higher number of affected joints.

Looking at the numerical differences of swollen and tender joint count, we found a mean difference of 2.6 (+ 3.6) joints for swelling and 2.0 (+ 3.3) for tenderness between patient and observer derived joint counts. Importantly, the discordance increased with the overall number of involved joints (Figure 3), showing a significant correlation (*P *< 0.01) between the numerical difference of swollen and tender joint count with SJC28 (r = 0.49) or respective TJC28 (r = 0.46).

Based on these findings and in line with our main hypothesis, we next performed the analyses in subgroups of patients in different states of disease activity according to SDAI (REM, *n *= 37; LDA, *n *= 100; and MDA/HAD, *n *= 72). Significant differences between the three subgroups for the mean divergences of patient and observer derived joint counts were found, namely a lower discrepancy for patients in REM compared with those in LDA, MDA/HDA (mean differences + SD, SJC: REM = 0.32 + 0.47; LDA = 2.0 + 2.3; MDA/HDA = 4.5 + 4.8, *P *< 0.001); TJC: REM = 0.38 + 0.68; LDA = 1.8 + 3.0; MDA/HDA = 3.1 + 4.0, *P *< 0.001;

Using Spearman correlation, we investigated which variables might influence the discordance and found a significant correlation of HAQ (r = 0.23), visual analogue score (VAS) for pain (r = 0.27), disease duration (r = 0.16) and SDAI (r = 0.55) with the mean discrepancy of swollen joint counts; and HAQ (r = 0.32), VAS pain (r = 0.39) and SDAI (r = 0.38) with discrepancy of tender joint counts. In a general linear model accounting for these variables we could show a linear increase of discrepancy of tender and swollen joint counts with increasing disease activity (mean differences (95%CI): SJC: REM = 0.68 (-0.5 to 1.9); LDA = 2.1 (1.5 to 2.8); MDA/HDA = 4.4 (3.5 to 5.3) *P *< 0.001; TJC: REM = 0.9 (-0.2 to 2.0); LDA = 2.0 (1.4 to 2.6); MDA/HDA = 3.1 (1.7 to 3.4) *P *< 0.001)

Using the Wilcoxon test, we found a high concordance of the joint counts obtained by patients and observer among patients in REM (SJC ties 25/37, TJC ties 26/37) whereas in LDA or MDA/HDA there was only a low frequency of exact matches (LDA: SJC ties 25/100, TJC ties 34/100; MDA/HDA: SJC ties 9/72, TJC ties 21/72).

Thus, since the major prerequisite for monitoring disease activity using patient self-assessed joint counts ought to be a high sensitivity compared to the assessment by a health professional, self-evaluation of swollen and tender joints should only be performed by patients in REM or at most LDA.

### Self assessed joint counts in determining the presence of remission

Table 3 shows the consistency of fulfilling the new ACR/EULAR Remission Definitions [7] when patient or biometrician joint assessments are used. The positive predictive value (PPV) of patient assessed remission for the different options suggested by the ACR/EULAR as the gold standard ranged between 0.71 and 0.86, the negative predictive value was even higher (0.85 to 0.95; Table 3).

**Table 3:** Agreement of patient defined remission with remission according to the ACR-EULAR definitions using the traditional evaluator derived joint counts (REF) ('gold standard').

	Boolean based without CRP		Boolean based with CRP		Index based CDAI*		Index based SDAI*	
	
	Patient derived REM	Gold standard REM	Patient derived REM	Gold standard REM	Patient derived REM	Gold standard REM	Patient derived REM	Gold standard REM
Patients in REM	*N *= 25	*N *= 34	*N *= 22	*N *= 28	*N *= 30	37	32	37
Patients not in REM	*n *= 171	*N *= 175	*N *= 177	*N *= 181	*N *= 147	172	164	172
Positive Predictive Value	**0.74**		**0.71**		**0.81**		**0.86**	
Negative Predictive Value	**0.98**		**0.98**		**0.85**		**0.95**	

CDAI, clinical disease activity index; REM, remission; SDAI, simplified disease activity index.

## Discussion

In this study we demonstrated that self assessed joint counts may be very useful in patients with RA who have very low levels of disease activity. This becomes especially important in the conflict between calls for tight control of RA on the one hand [3,4,13] and availability of resources on the other: accurate assessment especially of swollen and tender joints is time consuming and as physicians are facing a higher number of patients in routine care, patient self derived joint counts may constitute a helpful tool to allow for frequent assessment of disease activity without consuming health care capacities.

Several published studies showed different levels of agreement between patient and objective assessor derived joint counts. Our results suggest an overall moderate agreement of patient and observer derived joint counts, as expected from the literature [10-12]. Higher agreement was observed for the assessment of joint tenderness compared to joint swelling, also consistent with findings of previously reported studies [11]. Importantly, however, the finding that the agreement improves as the number of affected joints decreases is novel and may have crucial corollaries: when we specifically looked at patients in REM we found a very low numerical discrepancy between patient and observer derived joint counts (0.30 + 0.46), showing that patients in a good disease activity state were able to assess their disease activity as accurately as an expert evaluator, likely because in a good clinical state there is less room for a numerical error. Consequently, patients who rated themselves as being in remission by using their joint counts for definitions of remission had a high positive predictive value to be effectively in remission according to the new ACR/EULAR remission definitions. In this group of patients self derived assessment of disease activity seems to be a reliable and valid tool and, therefore, less frequent physician contacts would be feasible. These findings were independent of potential confounders, such as disease duration, pain or functional disability.

A simple training session did not substantially improve the agreement of patient and observer derived joint counts. While this may be surprising at first sight, there is evidence that even among trained health professionals with experience in assessing joints a substantial variation occurs [14,15]. Thus, our data suggest that there is not much benefit of formal training; nevertheless, it may be useful to instruct patients on the distinction between inflammatory and non-inflammatory joint swelling.

One strength of our study is the focus on real life patients with a broad range of disease activity and disease duration reflecting daily routine situations. This allowed us to investigate the main hypothesis of the present study, whether the reliability of patient-reported joint counts, which is viewed controversially in the literature, would be more convincing in low disease activity or remission than generally. Since these low disease activity states are now the treatment targets in RA [7,16], one would hope to see increasing numbers of patients in these good states. By having patients themselves assess the persistent absence of joint swelling the tight control concept could be further strengthened without overwhelming the available resources. This is also important in terms of pain as in low disease activity and remission patients frequently have no pain. If so, patient should be able to distinguish whether pain is due to swelling and 'flair' of RA and, therefore, call a rheumatologist or if pain is due to other problems one should visit their family doctor instead. On the other hand, the data presented also reveal that at higher states of disease activity major discrepancies can be discerned between patient and trained assessor derived joint counts. The high agreement in joint counts obtained by physicians and biometricians with similar disagreement compared with patient derived joint counts indicates that, with active disease, visits to a rheumatologist are indispensable.

Our study has several limitations. Firstly, the patient population is derived from a single center and, therefore, it is not self-evident that the data presented can be generalized. Ultimate proof or disproof will have to come from studies performed in other cohorts. Secondly, the analyses performed, even if evaluating two consecutive visits, were cross sectional in terms of treatments since therapeutic changes were not studied and consequent outcomes not assessed using patient derived joint counts. Thirdly, a possible gold standard related to the 'truthfulness' of patient versus assessor performed joint counts, such as by sonography or magnetic resonance imaging, was not performed and, therefore, it is not clear which of the counts is nearer to the 'truth', the presence or absence of synovial inflammation. In our study, we used the biometrician derived joint counts as a gold standard as they were performed by a trained health professional who assesses joints in daily routine. These joint counts are used for composite scores at our clinics. Furthermore we did not perform training sessions and assessed accuracy as addressed by Gall *et al. *[17] which might have improved the effect of training. Fourthly, joint assessments by different investigators were not randomized which might bias the results as showing constantly higher or lower joint counts at different assessments. Fifthly, since joint damage is correlated with swollen joints [1,18,19] and we did not assess radiographs, it is unclear which of the joint counts, patient or assessor derived, is more closely related to the progression of joint damage. These questions will have to be addressed in future studies and form an important research agenda.

## Conclusions

Self-assessed joint counts by patients who are in clinical remission are reliable and could be a useful tool in daily clinical practice as they allow effective follow up without need for tightly scheduled physician contacts. The data indicate a better level of agreement in joint assessment by patients in remission and with very low joint counts than in patients with more active disease. Although patient's self-assessment seems to be less effective in active disease, our data indicate that patients should be well able to confirm the maintenance of the previously observed state of remission over time. Therefore, patient reported disease activity should be incorporated in their daily routine for patients who have reached good clinical states as this could guide physicians in optimizing treatment strategies, while patients' self-performed joint counts in the presence of high disease activity cannot replace clinical assessment by the rheumatologist which, in these situations, therefore is indispensable.

## Abbreviations

ACR: American College of Rheumatology; CDAI: clinical disease activity index; CRP: C-reactive protein; DAS28: disease activity index 28 joints; EGA: evaluator global assessment of disease activity; ESR: erythrocyte sedimentation rate; EULAR: European League against Rheumatism; HAQ: health assessment questionnaire; HDA: high disease activity; ICC: intraclass correlation coefficient; MCP: metacarpo-phalangeal joint; MDA: moderate disease activity; LDA: low disease activity; PGA: patient global assessment of disease activity; PIP: proximal interphalangeal joint; RA: rheumatoid arthritis; REM: remission; SDAI: simplified disease activity index; SJC: swollen joint count; TJC: tender joint count; VAS: visual analogue scale.

## Competing interests

The authors declare that they have no competing interests.

## Authors' contributions

HR gave training sessions to the patients, performed joint counts, made statistical analyses, and wrote the first draft of the manuscript. JG, JS, TS and DA contributed to the final version of the manuscript by providing comments and suggestions on the statistical analyses and draft version of the manuscript. All authors read and approved the final manuscript.
